# Integrating Donor Derived Cell-Free DNA Fraction and Absolute Quantification for Enhanced Rejection Diagnosis in Kidney Transplant Recipients

**DOI:** 10.3390/diagnostics15030237

**Published:** 2025-01-21

**Authors:** Weijian Nie, Yan Wang, Qian Fu, Chenglin Wu, Ronghai Deng, Xiaolin Yu, Caiguo Ye, Xiangjun Liu, Bowen Xu, Pingping Sun, Longshan Liu, Jun Li, Huanxi Zhang, Changxi Wang

**Affiliations:** 1 Organ Transplant Center, The First Affiliated Hospital, Sun Yat-sen University, Guangzhou 510080, China; weacandnie@163.com (W.N.); qf902@163.com (Q.F.); wuchlin7@mail.sysu.edu.cn (C.W.); dengrh3@mail.sysu.edu.cn (R.D.); xubw8@mail.sysu.edu.cn (B.X.); liulshan@mail.sysu.edu.cn (L.L.); wangchx@mail.sysu.edu.cn (C.W.); 2 Department of Medical Ultrasonics, Institute of Diagnostic and Interventional Ultrasound, The First Affiliated Hospital, Sun Yat-sen University, Guangzhou 510080, China; wyan29@mail.sysu.edu.cn; 3 Guangzhou Bo Fu Rui Medical Laboratory Co., Ltd., Guangzhou 510080, China; xlyu@bfrbiotech.com.cn (X.Y.); cgye@bfrbiotech.com.cn (C.Y.); xjliu@bfrbiotech.com.cn (X.L.); 4 GCP Office, The First Affiliated Hospital, Sun Yat-sen University, Guangzhou 510080, China; sunpp3@mail.sysu.edu.cn; 5 Guangdong Provincial Key Laboratory of Organ Medicine, The First Affiliated Hospital, Sun Yat-sen University, Guangzhou 510080, China; 6 Guangdong Provincial International Cooperation Base of Science and Technology (Organ Transplantation), The First Affiliated Hospital, Sun Yat-sen University, Guangzhou 510080, China

**Keywords:** kidney transplantation, cell-free DNA, rejection, diagnosis

## Abstract

**Background:** This study aimed to assess the diagnostic accuracy of combining donor-derived cell-free DNA (dd-cfDNA) fraction and absolute quantification in detecting kidney allograft rejection. **Methods:** A prospective study was conducted from December 2019 to April 2021 at the First Affiliated Hospital of Sun Yat-sen University. Kidney transplant recipients who underwent biopsy, including cases of T-cell-mediated rejection (TCMR), antibody-mediated rejection (ABMR), and borderline rejection, were included. dd-cfDNA fraction and absolute concentrations were measured, and diagnostic efficacy was evaluated using receiver operating characteristic (ROC) analysis. The double-positive and double-negative methods were applied to assess performance. **Results:** A total of 50 kidney transplant recipients were included. The dd-cfDNA fraction cutoff of 1.08% achieved 93.33% sensitivity and 91.43% specificity (AUC = 0.95), with an NPV of 96.97% and a PPV of 82.35%. The absolute dd-cfDNA threshold of 32 cp/mL yielded 80.00% sensitivity and 71.43% specificity (AUC = 0.78), with an NPV of 89.29% and a PPV of 54.55%. The double-positive method provided superior accuracy, with a PPV of 91.67% and an NPV of 89.47%, demonstrating 73.33% sensitivity and 97.14% specificity. The double-negative method achieved 100% NPV and 100% sensitivity. **Conclusions:** Combining dd-cfDNA fraction and absolute quantification improves diagnostic accuracy for kidney transplant rejection, especially ABMR. The double-positive and double-negative approaches show high predictive value, offering potential clinical value for monitoring kidney transplant recipients.

## 1. Introduction

Kidney transplantation (KTx) is the preferred treatment for patients with end-stage renal disease. Despite significant advancements in surgical techniques and immunosuppressive therapies, which have improved graft function and extended patient survival, immune rejection remains a critical challenge in allogeneic transplantation. Globally, around 12% of patients experience acute rejection within the first year post-transplant, and long-term outcomes show that the 10-year survival rate of renal allografts drops to 55% [1,2].

To monitor graft complications in KTx recipients, several non-invasive diagnostic methods, such as plasma creatinine, proteinuria, donor-specific antibodies (DSA), and ultrasonography, are commonly employed [3]. However, these approaches often lack sufficient sensitivity and specificity. Recently, donor-derived cell-free DNA (dd-cfDNA) testing has gained traction as a non-invasive method to detect early graft injury, potentially reducing the need for unnecessary biopsies triggered by elevated plasma creatinine levels [2,4]. Since organ transplantation essentially transfers the donor’s genome into the recipient, dd-cfDNA serves as a marker of allograft cell death, offering a sensitive indication of graft injury, including rejection [5]. Detected in both urine and blood, dd-cfDNA has shown promise as a non-invasive biomarker for diagnosing allograft rejection [5,6] and is now being implemented in clinical settings in some transplant centers.

In previous studies, dd-cfDNA quantification has been expressed as both the ratio of donor-derived sequences to total cfDNA (ND/N) and as an absolute copy number (copies/mL of plasma) [7,8]. While each method has its advantages, both also have limitations. Total plasma cfDNA reflects the recipient’s physiological state, and increases in cfDNA can be observed in non-rejection-related conditions such as sepsis, stroke, infarction, or malignancy [9,10]. Therefore, the dd-cfDNA ratio may be influenced by these factors, complicating its interpretation in isolation [2]. Furthermore, cfDNA released from apoptotic and necrotic cells is often cleaved into smaller fragments, and dd-cfDNA from solid organs has been shown to be shorter than cfDNA from recipient white blood cells. This may lead to underestimation of dd-cfDNA levels when using PCR-based methods, which are commonly employed for cfDNA detection due to their lower cost and widespread use [11].

Given the limited research that has simultaneously applied both dd-cfDNA ratio and absolute quantification methods for rejection detection, our study seeks to fill this gap by investigating whether their combined use enhances diagnostic accuracy. We hypothesize that integrating these two approaches will provide superior results compared to using either method alone in diagnosing kidney transplant rejection.

## 2. Materials and Methods

### 2.1. Study Design

This prospective study aimed to evaluate the diagnostic accuracy of the dd-cfDNA ratio and absolute quantification in detecting kidney allograft rejection. Kidney transplant recipients at the First Affiliated Hospital of Sun Yat-sen University from December 2019 to April 2021 were screened for eligibility. Inclusion criteria included patients who underwent a kidney allograft biopsy after kidney transplantation. Exclusion criteria were as follows: (1) multiorgan or prior organ transplantation, (2) cancer or pediatric patients, (3) within 14 days post-transplant, and (4) patients who declined to provide consent. The study was conducted in accordance with the Declaration of Helsinki and received approval from the Institutional Review Board of the First Affiliated Hospital of Sun Yat-sen University.

### 2.2. Histological Diagnosis

Pathological biopsy results were evaluated by pathologists from the Department of Pathology at the First Affiliated Hospital of Sun Yat-sen University, following the latest Banff 2019 guidelines [12]. Both pathologists and researchers were blinded to the dd-cfDNA results. Patients were classified with antibody-mediated rejection (ABMR), T-cell-mediated rejection (TCMR), mixed rejection, BK virus nephropathy (BKVN), focal segmental glomerulosclerosis (FSGS), IgA nephropathy (IgAN), or other nonspecific conditions. ABMR, TCMR, and mixed rejection were grouped as the rejection category, while borderline rejection, BKVN, FSGS, IgAN, and other nonspecific conditions formed the non-rejection group. 

### 2.3. Blood Sample Collection and cfDNA Extraction

For each patient, 10 mL of peripheral blood was drawn into Cell-Free DNA BCT collection tubes (Streck, La Vista, NE, USA). The samples were first centrifuged at 2000 rpm for 15 min at 4 °C, and the plasma was carefully collected. A second centrifugation was performed at 13,000 rpm for 5 min at 4 °C to ensure the removal of any remaining cellular components. cfDNA was then isolated from the plasma using the Magnetic Serum/Plasma DNA Maxi Kit (TIANGEN, Beijing, China), following the manufacturer’s instructions.

### 2.4. Quantification of cfDNA

Quantification of total cfDNA was performed using quantitative polymerase chain reaction (qPCR) on two Alu repetitive sequences with an Applied Biosystems 7500 Real-Time PCR System (Thermo Fisher Scientific, Waltham, MA, USA). Two sets of primers were employed: the first set used 5′-CCTGAGGTCAGGAGTTCGAG-3′ (forward) and 5′-GCCCCGGCTAATTTTTGTAT-3′ (reverse) to generate an 81 bp amplicon; the second set used the same forward primer with 5′-CCCGAGTAGCTGGGATTACA-3′ (reverse) to produce a 115 bp amplicon. A standard curve was established using serial 1:10 dilutions of commercial human genomic DNA purified from the buffy coat (Roche, Basel, Switzerland).

Each qPCR reaction was set up in triplicate, with 20 µL reaction volumes containing 2 µL of cfDNA (equivalent to 2 µL of plasma), 0.2 µM forward and reverse primers, 0.4 µL ROX Reference Dye (Thermo Fisher Scientific, Waltham, MA, USA), 1.2 µL DMSO (Sigma-Aldrich, St. Louis, MO, USA), and qPCR Master Mix (Thermo Fisher Scientific, Waltham, MA, USA). The cycling conditions were 3 min at 95 °C, followed by 45 cycles of 95 °C for 15 s, 62 °C for 30 s, and 50 °C for 1 min. The cfDNA concentration for each sample was calculated by averaging the values obtained from both primer pairs.

### 2.5. SNP Selection, Targeted Sequencing, and Data Analysis

To make the dd-cfDNA assay universally applicable to transplant recipients without requiring separate genotyping of either the donor or recipient, a selection of single nucleotide polymorphisms (SNPs) was made to ensure that the same SNP panel could be used across individuals in the Chinese population. A total of 441 polymorphic SNPs were selected, sufficient to differentiate between any two unrelated individuals. These SNPs have an average heterozygosity > 0.4. Probes targeting these SNPs were designed to be 78 nucleotides in length [13].

Targeted libraries were constructed using the KAPA Hyper Library Construction Kit (KAPA Biosystems, Wilmington, MA, USA), and DNA sequencing was performed on an Illumina MiSeq sequencer (Illumina, San Diego, CA, USA). SNP calling followed published protocols, utilizing a series of software tools for sequencing data analysis. First, sequencing adapters were removed using Cutadapt (version 1.16), and low-quality bases were filtered to generate clean reads. These clean reads were then aligned to the human reference genome (hg19) using BWA (version 0.7.12-r1044).

To further process the data, Bamtools was employed to filter out unqualified sequences, while Bammarkduplicates2 was used to remove duplicate reads. The GATK ApplyBQSR module was applied to recalibrate base quality scores within the BAM files. Variant identification, including SNPs and insertions/deletions (InDels), was performed using GATK Mutect2, followed by GATK FilterMutectCalls to filter out variants potentially arising from contamination, ensuring high-quality mutation data.

### 2.6. dd-cfDNA Quantification

Plasma dd-cfDNA levels were quantified using a targeted next-generation sequencing (NGS) assay that employed a 441 SNP panel. This panel demonstrated a high degree of heterozygosity, with a minor allele frequency (MAF) > 0.4 in the Chinese population, and was adapted based on a previously published method [13]. The dd-cfDNA fraction was calculated using a maximum likelihood estimation-based approach to ensure accuracy in quantification.

(1) $$L(\theta )=\prod_{i=1}^{n}p\;(\chi_{i}|\theta )$$

The value of $\chi_{i}$ for a specific informative SNPi is calculated as follows:

$$\chi_{i}=p_{hom}\times a_{i}+p_{het}\times 2a_{i}$$

where $a_{i}$ represents the minor allele counts divided by the total allele counts for the SNP, $p_{hom}$ is the homozygous frequency of the minor allele in the Chinese population, and $p_{het}$ is the heterozygous frequency of the minor allele in the Chinese population. The likelihood function L(θ) is then transformed to l(θ):

(2) $$l(\theta )=\sum \ln p\;(\chi_{i}|\theta )$$

The maximum likelihood estimate of $l\left(\theta \right)$ was determined using the general optimizer nlminb () function in R (https://www.r-project.org/). The dd-cfDNA absolute quantification was then calculated using the following formula:

dd-cfDNA absolute quantification = total cfDNA × dd-cfDNA fraction

### 2.7. Statistical Analysis

Continuous data were expressed as the median with interquartile ranges (IQR), while categorical data were presented as frequencies. For comparisons of continuous variables, Student’s *t*-test was used when the assumption of normality was met, and the Mann–Whitney U-test was applied for non-normally distributed data. Categorical variables were compared using either chi-square tests or Fisher’s exact tests, depending on the data distribution. The receiver operating characteristic (ROC) curve was employed to assess the diagnostic accuracy of both dd-cfDNA fraction and absolute quantification in identifying renal transplant rejection. All statistical analyses were conducted using IBM SPSS software, version 22 (IBM Corporation, Armonk, NY, USA). Graphs were generated with GraphPad Prism 9 and MedCalc Statistical Software, version 20.218.

## 3. Results

### 3.1. Patient Cohort and Characteristics

A total of 50 patients were recruited and analyzed in this study. The key characteristics of the recipients are summarized in Table 1. Plasma creatinine concentration, commonly used as a diagnostic marker for kidney dysfunction and a trigger for biopsy, did not show a significant difference between the rejection and non-rejection groups (*p* = 0.719). Similarly, no differences were observed in the estimated glomerular filtration rate (eGFR) between the two groups (*p* = 0.857).

The standard immunosuppressive regimen, consisting of calcineurin inhibitors (CNI), mycophenolic acid (MPA), and steroids, was used in both groups. The proportion of patients using tacrolimus did not differ significantly between the rejection and non-rejection groups (*p* = 1.00). Additionally, the type of organ donation was not identified as a risk factor for kidney rejection. There were no significant differences between the two groups in terms of age, sex, white blood cell count, or cold ischemia time.

### 3.2. dd-cfDNA Fraction and Absolute Quantification Among Groups

Based on laboratory and pathological diagnoses, 14 patients were identified with ABMR, 1 with TCMR, and 6 with borderline TCMR. The remaining patients were diagnosed with BKVN, IgAN, FSGS, or unspecified kidney diseases (Table 2). For analysis, the 14 ABMR patients and 1 TCMR patient were grouped into the rejection group, while the remaining patients were classified as the non-rejection group.

**Figure 1:**
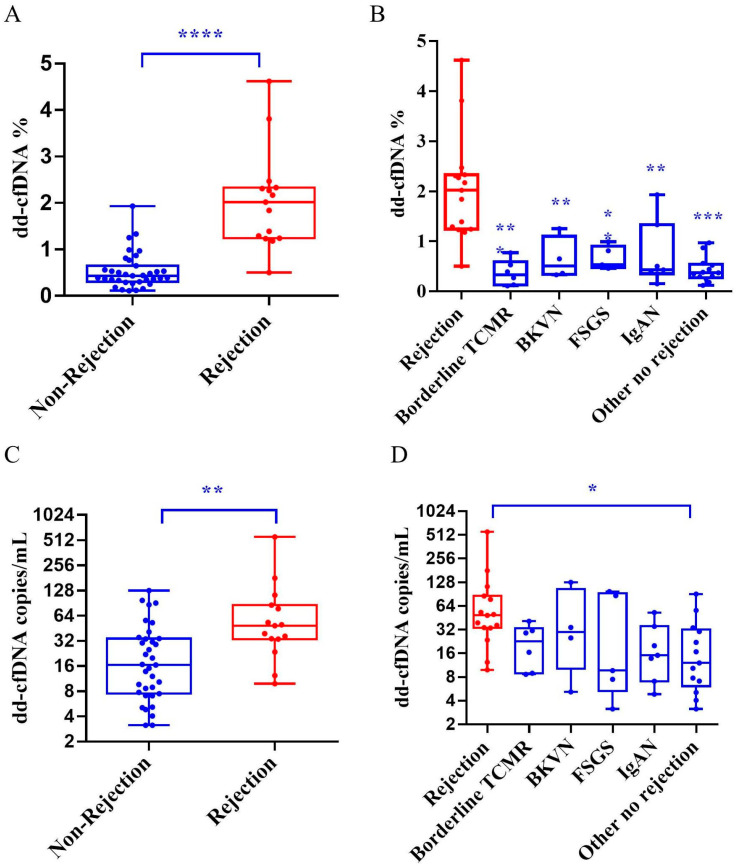
Comparison of dd-cfDNA level across groups. dd-cfDNA fraction in the rejection and non-rejection groups ((**A**) **** *p* < 0.0001), with detailed subgroup analysis including borderline TCMR, BKVN, IgAN, and FSGS ((**B**) * *p* < 0.05, ** *p* < 0.01, *** *p* < 0.001). dd-cfDNA absolute quantification in the rejection and non-rejection groups ((**C**) ** *p* < 0.01), with subgroup comparisons for borderline TCMR, BKVN, IgAN, and FSGS ((**D**) * *p* < 0.05).

The results showed that the median dd-cfDNA fraction in the rejection group was 2.02% (Q1–Q3, 1.24–2.33%) compared to 0.43% (Q1–Q3, 0.30–0.65%) in the non-rejection group (Figure 1A, *p* < 0.01). Interestingly, the dd-cfDNA fraction in the borderline TCMR group was significantly lower than in the rejection group and closely resembled the levels found in the BKVN, IgAN, and FSGS groups (Figure 1B, *p* < 0.01).

We also analyzed absolute dd-cfDNA concentrations using the absolute copy number method. The median copy number in the rejection group was 49 copies/mL (Q1–Q3, 33–85), while in the non-rejection group it was 16 copies/mL (Q1–Q3, 7–34) (Figure 1C, *p* < 0.01). In subgroup comparisons, the borderline TCMR group showed similar dd-cfDNA copy numbers to the BKVN, IgAN, and FSGS groups. Only in the non-specific kidney disease group was the absolute dd-cfDNA concentration significantly lower than that of the rejection group (Figure 1D, *p* < 0.05).

These findings indicate that both the dd-cfDNA fraction and absolute dd-cfDNA concentrations are effective biomarkers, particularly for ABMR following kidney transplantation. However, the dd-cfDNA fraction demonstrated superior discriminatory performance compared to the absolute copy number method.

### 3.3. Diagnostic Performance for Kidney Allograft Rejection

**Figure 2:**
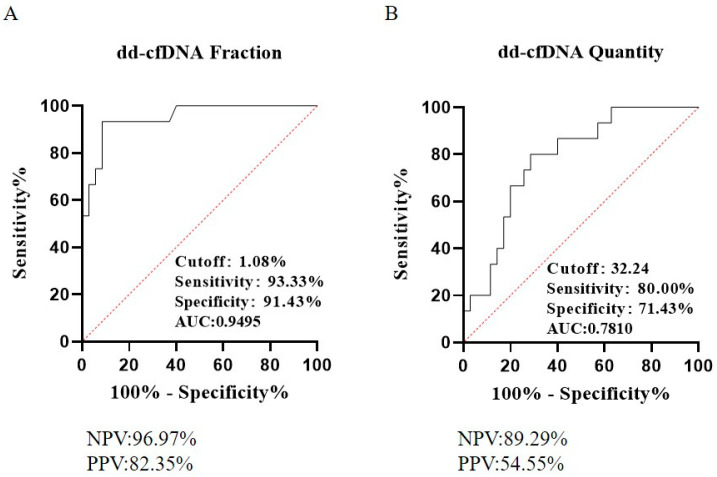
ROC curves for dd-cfDNA fraction (**A**) and absolute copy number (**B**) in differentiating between rejection and non-rejection groups. The optimal cutoff for the dd-cfDNA fraction is 1.08%, and for the absolute copy number, it is 32 cp/mL, both determined by the maximum Youden index.

**Figure 3:**
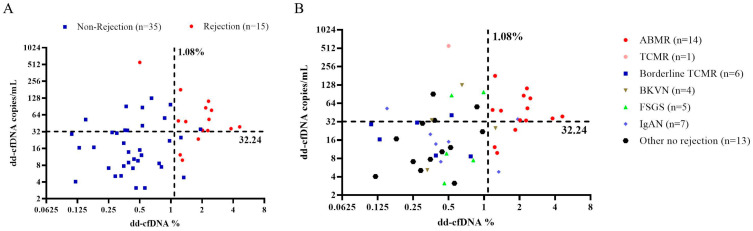
dd-cfDNA fraction and absolute copy number with thresholds set at 1.08% and 32 cp/mL, respectively, to distinguish between rejection and non-rejection groups (**A**). Subgroups including borderline TCMR, BKVN, IgAN, FSGS, and unspecified kidney diseases are labeled (**B**).

Using a dd-cfDNA fraction cutoff value of 1.08% to differentiate between rejection and non-rejection groups through ROC analysis, we achieved a sensitivity of 93.33% and a specificity of 91.43% (Figure 2A, AUC = 0.95). The negative predictive value (NPV) was 96.97%, and the positive predictive value (PPV) was 82.35%. In comparison, using the absolute copy number method with a threshold of 32 copies/mL resulted in a sensitivity of 80.00% and specificity of 71.43% (Figure 3B, AUC = 0.78). The corresponding NPV and PPV for the absolute method were 89.29% and 54.55%, respectively (Figure 2B).

### 3.4. Integrating dd-cfDNA Fraction and Absolute Quantification

The “double-positive” dd-cfDNA method, defined as positive for both the dd-cfDNA fraction and absolute quantification, significantly improved the PPV from 82.35% (fraction) and 54.55% (absolute) to 91.67% (Figure 3, Table 3, line 4). Only one false positive case was observed, diagnosed as IgAN (1/12) (Figure 3B, upper-right region). The method demonstrated a sensitivity of 73.33% and a specificity of 97.14% for diagnosing rejection (Table 3, line 4).

Similarly, the “double-negative” dd-cfDNA method, defined as negative for both the dd-cfDNA fraction and absolute quantification, increased the NPV from 96.97% (fraction) and 89.29% (absolute) to 100% (Figure 3A), with a sensitivity of 100% (Table 3).

## 4. Discussion

This study is one of the earliest to explore the combined use of both dd-cfDNA fraction and absolute quantification for diagnosing kidney transplant rejection. Among 50 kidney transplant recipients, our findings demonstrate that utilizing both parameters simultaneously—through a double-positive and double-negative approach—significantly enhances the positive predictive value (PPV) and negative predictive value (NPV) of the diagnostic process.

Our results align with recent studies that advocate for the combined assessment of relative and absolute dd-cfDNA levels to improve diagnostic accuracy. Osmanodja et al. [14], in their study involving 22 kidney transplant patients, suggested that employing both absolute and relative dd-cfDNA measurements enhances reliability and comparability among individuals in clinical settings. Similarly, Bunnapradist et al. [15] reported data from 41 kidney transplant patients and observed that relying solely on fractional dd-cfDNA could lead to false-negative results. They proposed a two-threshold algorithm combining both fractional dd-cfDNA (≥1%) and absolute dd-cfDNA (≥78 cp/mL), wherein samples exceeding both thresholds are considered at high risk for allograft rejection. This approach was shown to improve sensitivity in detecting acute rejection while maintaining high specificity.

Elevated dd-cfDNA levels have also been observed in patients with pyelonephritis and biopsy-proven BK virus nephropathy [3,16], indicating that dd-cfDNA can be influenced by factors other than rejection. The clinical validity of dd-cfDNA in kidney transplantation has been documented, with threshold values for fractional determination reported around 0.43% and appropriate absolute quantification at 52 cp/mL based on the simultaneous maximization of sensitivity and specificity [2]. Although different methodologies have been used to establish threshold values for rejection detection, median dd-cfDNA values in reference populations are remarkably similar, ranging from 0.21% to 0.40% [2,17]. In kidney transplantation, recent studies have reported lower limits for dd-cfDNA fractions as low as 0.08% [18], while in heart transplantation, thresholds range from 0.02% to 0.07% [19,20]. Many studies have demonstrated that the cutoff for fractional dd-cfDNA in biopsy-proven rejection patients ranges from 0.42% to 1% [2,4,15,21]. In a large-scale and critical review analysis of dd-cfDNA application in kidney damage and rejection prediction, the authors draw the conclusion that dd-cfDNA is a promising biomarker capable of predicting acute rejection organ transplants [22]. Definitely, among other biomarkers, Jimenez-Coll et al. concluded that dd-cfDNA could serve as an optimal marker of kidney damage [23]. In our study, using a dd-cfDNA fraction cutoff of 1.08%, we achieved a sensitivity of 93.33% and a specificity of 91.43% in diagnosing rejection. However, the fractional dd-cfDNA can be influenced by the total amount of cfDNA from the recipient, which may vary due to infection, exercise, medications, and other conditions [10]. Additionally, a time-dependent increase in dd-cfDNA fraction during long-term surveillance—from 0.8% to 2.1% (90th percentile)—has been observed concurrently with a decrease in total cfDNA [24]. This variability raises concerns about the sole reliance on fractional dd-cfDNA for rejection diagnosis.

In contrast, absolute dd-cfDNA quantification is less affected by changes in recipient cfDNA levels and has been validated using digital PCR methods [2,4]. Oellerich et al. [25] suggested that absolute dd-cfDNA quantification effectively discriminated among patients with T-cell-mediated rejection (TCMR), antibody-mediated rejection (ABMR), borderline TCMR, and acute tubular necrosis, compared to stable patients and those with negative biopsies. Some studies have reported that the diagnostic accuracy (AUC-ROC) for detecting acute rejection is superior when using absolute quantification (83%) compared to fractional dd-cfDNA determination (73%) [2]. In our study, the absolute dd-cfDNA threshold of 32 cp/mL yielded an NPV of 89.29% and a PPV of 54.55%, with a sensitivity of 80.00% and specificity of 71.43%. However, the diagnostic performance of absolute dd-cfDNA was lower than that of dd-cfDNA fraction (78% vs. 95%).

By combining both the dd-cfDNA fraction and absolute quantification in a “double-positive” method, we observed an improvement in PPV to 91.67% for kidney rejection diagnosis. Only one false-positive case was noted, diagnosed as IgA nephropathy. This approach underscores the enhanced diagnostic efficacy achieved by integrating both measurements, as also suggested by other studies. For instance, a smaller-scale study (*n* = 22) demonstrated that combining absolute and relative dd-cfDNA measurements improved reliability and interindividual comparability in chronic ABMR [14]. Similarly, another study (*n* = 41) reported that combining absolute and relative dd-cfDNA measurements significantly improved sensitivity and specificity for detecting kidney rejection [15]. Halloran et al. also demonstrated that the combination of dd-cfDNA fraction and quantity is more predictive than either value alone, achieving a sensitivity of 83.1% and specificity of 81.0% for molecular diagnoses [26]. Our findings, in agreement with previous findings, indicate that combining dd-cfDNA ratio and absolute value provides greater diagnostic value for kidney transplant rejection monitoring.

Despite these promising results, our study has several limitations. First, it is a prospective study without a validation cohort, which may limit the generalizability of the findings. Second, the sample size is relatively small, particularly in certain subgroups, with only one patient in the TCMR group and six in the borderline group. This limited sample size may restrict the robustness of conclusions drawn for these subtypes. While our findings provide preliminary insights, they should be interpreted with caution, and further studies with larger cohorts are necessary to confirm these observations and improve their generalizability. The observed false-negative results in TCMR and borderline TCMR cases may be due to insufficient detection of short DNA fragments, as our assay used relatively long amplicons (100–130 bp). Independent studies have confirmed that dd-cfDNA undergoes more extensive fragmentation in the kidney tubulointerstitial space in TCMR and borderline TCMR compared to ABMR [13,27], which may affect detection sensitivity.

## 5. Conclusions

In conclusion, our study demonstrates that the combined use of dd-cfDNA fraction and absolute quantification enhances the diagnostic accuracy for kidney transplant rejection. These findings support the implementation of combined dd-cfDNA measurements in clinical practice to improve the detection of acute rejection in kidney transplant recipients. 

## Figures and Tables

**Table 1:** Patients’ demographic and clinical characteristics.

Characteristics			Non-Rejection (*n* = 35)	Rejection (*n* = 15)	*p*
**Recipients Characteristics**					
Age (years, IQR)			35 (28–45)	43 (27–57)	0.268
Male sex (*n*, %)			27 (77.1%)	12 (80.0%)	1.000
Median time posttransplant (years, IQR)			1.16 (0.43–2.43)	6.17 (0.28–8.55)	0.035
Baseline characteristics at biopsy					
	Renal function				
		Creatinine (umol/L)	156 (112–247)	144 (110–256)	0.719
		eGFR (mL/min/1.73 m^2^, IQR)	54.81 (29.77–80.20)	60.04 (31.18–80.30)	0.857
	Urine protein				-
		0 (*n*, %)	21(60.0%)	7 (46.7%)	
		1+ (*n*, %)	8 (22.9%)	7 (46.7%)	
		2+ (*n*, %)	4 (11.4%)	1 (6.7%)	
		3+ (*n*, %)	2 (5.7%)	0 (0.0%)	
	White blood cell (×10^9^/L, IQR)		6.51 (5.61–8.52)	6.64 (4.99–7.86)	0.735
	Neutrophils (×10^9^/L, IQR)		4.96 (3.25–7.41)	4.20 (3.16–5.68)	0.816
	Cause for biopsy				-
		Raised creatinine (*n*, %)	17 (48.6%)	8 (53.3%)	
		Proteinuria (*n*, %)	8 (22.9%)	3 (20.0%)	
		Hematuria (*n*, %)	5 (14.3%)	0 (0.0%)	
		PRA Positive (*n*, %)	0 (0.0%)	3 (20.0%)	
		Others (*n*, %)	5 (14.3%)	1 (6.7%)	
	CNI				1.000
		Tacrolimus (*n*, %)	32 (91.4%)	14 (93.3%)	
		Cyclosporin (*n*, %)	3 (8.6%)	1 (6.7%)	
	CNI trough concentration				
		Tacrolimus (ng/mL, IQR)	7.10 (6.05–8.35)	5.80 (4.18–7.88)	0.471
		Cyclosporin (ng/mL, IQR)	85.80 (68.00–)	49.30–	-
	DSA positive (*n*, %)		2 (5.7%)	13 (86.7%)	0.000
**Donor Characteristics**					
Donor type					1.000
	Deceased-donor (*n*, %)		28 (80.0%)	12 (80.0%)	
	Living-donor (*n*, %)		7 (20.0%)	3 (20.0%)	

Abbreviation: CNI, calcineurin inhibitor; DSA, donor-specific antibody.

**Table 2:** Comparison of dd-cfDNA fraction and absolute quantification across groups.

All Recipients (*n* = 50)			
Banff Histology	Frequency (*n*, %)	dd-cfDNA Fraction (%, Q1–Q3)	dd-cfDNA Absolute Quantification (copies/mL, Q1–Q3)
ABMR *	14 (28.0%)	2.10 (1.28–2.36)	43.73 (31.02–79.40)
TCMR	1 (2.0%)	0.50–	557.55–
Borderline TCMR	6 (12.0%)	0.33 (0.13–0.59)	22.72 (8.85–33.47)
BKVN	4 (8.0%)	0.51 (0.34–1.10)	29.56 (10.14–104.07)
IgAN/FSGS	12 (24.0%)	0.49 (0.40–0.95)	14.42 (7.17–48.32)
Other no rejection	13 (26.0%)	0.37 (0.27–0.54)	12.08 (6.10–32.00)

* ABMR, antibody-mediated rejection and mixed rejection.

**Table 3:** Comparison of diagnostic performance.

	Cut-Off Value (%, Copy Number)	PPV	NPV	Sensitivity	Specificity
dd-cfDNA Fraction	1.08	82.35%	96.97%	93.33%	91.43%
dd-cfDNA copy number	32	54.55%	89.29%	80.00%	71.43%
Double-negative	1.08,32	55.56%	100.00%	100.00%	65.71%
Double-positive	1.08, 32	91.67%	89.47%	73.33%	97.14%

Abbreviation: PPV, positive predicted value; NPV, negative predicted value.

## Data Availability

The data presented in this study are available on request from the corresponding author. Access to the data is restricted due to patient privacy and regulations set by the ethics committee. Data will be shared only for research purposes upon appropriate authorization.
